# Exploring the characteristics of gut microbiota in the development and progression of early-stage colorectal cancer based on metagenomic sequencing

**DOI:** 10.3389/fmicb.2025.1658160

**Published:** 2025-11-20

**Authors:** Qianqian Chen, Jing Guan, Lu Yang, Jie Lv, Gen Gui, Jianhua Xu, Zhaoyun Yang, Xu Wang, Bin Sun

**Affiliations:** 1 Department of Gastroenterology, The First Affiliated Hospital of Anhui Medical University, 218 Jixi Road, Hefei, Anhui, China; 2 Anhui Provincial Key Laboratory of Digestive Disease, The First Affiliated Hospital of Anhui Medical University, Hefei, Anhui, China

**Keywords:** colorectal cancer, colorectal adenomas, gut microbiota, intramucosal carcinoma, polyps

## Abstract

**Introduction:**

Colorectal cancer (CRC), a leading cause of cancer-related morbidity and mortality worldwide, often presents asymptomatically, resulting in late diagnosis. Accumulating evidence links gut microbiota dysbiosis to CRC initiation and progression.

**Objective:**

This study aimed to investigate the differences in gut microbiota composition and diversity among healthy controls (HC) and patients with colorectal lesions—including common colorectal polyps, small colorectal adenomas (SCRA), large colorectal adenomas (LCRA), and intramucosal carcinoma (IMC)—to identify bacterial species associated with disease progression and provide novel insights into the diagnosis and treatment of CRC based on the “polyp-adenoma-carcinoma” sequence.

**Methods:**

A total of 250 participants were recruited from the First Affiliated Hospital of Anhui Medical University between July 2023 and June 2024. The cohort included 30 HC, 52 with common colorectal polyps, 58 with SCRA, 56 with LCRA, and 54 with IMC. Fecal samples were collected for bacterial DNA extraction, followed by metagenomic sequencing to analyze microbial diversity. Differential microbiota analysis was performed using the R package microbiomeMarker and LEfSe. Group classification and feature identification were conducted using a random forest model. Functional profiling was performed using DIAMOND against the KEGG and MetaCyc databases.

**Results:**

No significant differences in *α*-diversity were observed across the groups. *β*-diversity analysis revealed significant differences in Bray-Curtis and Jaccard distances among the groups. The composition and abundance of gut microbiota at the phylum, class, order, family, genus, and species levels were significantly altered. LEfSe analysis identified specific bacterial species with significant differences in IMC compared to other groups. Furthermore, the random forest model effectively distinguished patients with IMC from other groups based on distinct microbial signatures. Functional profiling revealed that the gut microbiota undergoes metabolic reprogramming from a homeostatic to a pro-tumorigenic phenotype during CRC progression as well as reduced protective pathway abundance and impaired energy/biosynthetic metabolism in CRC-associated microbiota.

**Conclusion:**

Gut microbiota profiles varied significantly among HC, polyp, SCRA, LCRA, and IMC groups. Specific microbial signatures were able to effectively differentiate IMC from both HC and non-malignant colorectal lesions, highlighting their potential as diagnostic biomarkers.

## 1 Introduction

Colorectal cancer (CRC) is the third most commonly diagnosed malignancy and the second leading cause of cancer-related mortality worldwide (Siegel et al., 2024). Its initiation and progression result from complex interactions among genetic, environmental, and microbial factors (Thulasinathan et al., 2025). Recent research has emphasized the role of the gut microbiota—often referred to as the “hidden organ”—as a critical breakthrough in CRC studies due to its involvement in host metabolism, immune regulation, and the maintenance of mucosal integrity (Thulasinathan et al., 2025; Huang and Huang, 2022). Mounting evidence indicates that dysbiosis of the gut microbiota is not merely a consequence but may be a pivotal factor contributing to carcinogenesis (Huang and Huang, 2022; Roy et al., 2025).

Advances in metagenomic sequencing (MS) and metabolomics have increasingly linked abnormalities in gut microbiota composition and function to the development of colorectal diseases (Hemmati et al., 2024). MS, which sequences the genomes of all microorganisms in intestinal contents, provides comprehensive insights into microbial species, gene functions, and metabolic pathways (Fusco et al., 2023). Compared to traditional 16S rRNA sequencing, MS provides strain-level resolution, functional gene annotation and novel taxon discovery capabilities, enabling comprehensive profiling of microbial communities (Wang et al., 2023; Ionescu et al., 2023; Lei et al., 2024).

Substantial evidence implicates the gut microbiota as a critical mediator in the progression from benign polyps to adenomatous lesions and ultimately CRC (Ocvirk and O'Keefe, 2021). However, most current research focuses on characterizing microbial changes in established CRC, with limited understanding of the microbial dynamics during the precancerous stages—specifically, the progression from benign polyps to adenomas and eventually to carcinoma.

The development of CRC typically follows the classical “adenoma-carcinoma” sequence, a multistage progression that provides a unique opportunity to explore the dynamic evolution of gut microbiota (Ocvirk and O'Keefe, 2021). In healthy individuals, the gut microbiota exhibits high diversity and mutualistic balance. However, as mucosal lesions evolve from common colorectal polyps to SCRA, LCRA, and ultimately IMC, probiotics such as butyrate-producing bacteria decline while pro-inflammatory taxa expand (Thulasinathan et al., 2025; Ocvirk and O'Keefe, 2021). These alterations suggest the potential utility of microbial markers for early detection and provide a theoretical basis for microbiota-targeted interventions aimed at delaying or preventing CRC onset.

Nevertheless, stage-specific microbial characteristics across the spectrum from healthy controls to various stages of colorectal lesions—including polyps, SCRA, LCRA, and IMC—remain inadequately elucidated. In this study, we systematically evaluated gut microbiota composition and structural variation among these groups to characterize the microbial successional trajectory during colorectal carcinogenesis. Our goal was to provide new perspectives for early diagnosis, risk stratification, and targeted microbial interventions in CRC prevention and management based on the “polyp-adenoma-carcinoma” continuum.

## 2 Materials and methods

### 2.1 Human subjects

This study prospectively enrolled 250 participants at the Department of Gastroenterology, The First Affiliated Hospital of Anhui Medical University, between July 2023 and June 2024. Based on colonoscopic and histopathological evaluations, individuals were categorized into the following groups: healthy controls (HC), common colorectal polyps (Polyp), small colorectal adenomas (SCRA), large colorectal adenomas (LCRA), and colorectal intramucosal carcinomas (IMC). Mid-portion fecal samples were collected using standardized stool collection kits prior to bowel preparation for colonoscopy and were preserved at −80 °C within 2 hours of collection.

Disease classification and diagnostic criteria were based on multiple authoritative guidelines, including the Standardized Diagnosis and Treatment of Colorectal Polyps, the Chinese Guidelines for the Diagnosis and Treatment of Colorectal Cancer (2023 Edition), the American Joint Committee on Cancer (AJCC) 8th Edition, and the Chinese Guidelines for Screening, Early Diagnosis, and Early Treatment of Colorectal Cancer (2020 Beijing Edition) (Wang et al., 2024; National Health Commission of the People's Republic of China, 2023; Giuliano et al., 2018; National Cancer Center, China, Expert Group of the Development of China Guideline for the Screening, Early Detection and Early Treatment of Colorectal Cancer, 2021). Specifically, common colorectal polyps were defined as hyperplastic or inflammatory polyps. SCRA were defined as tubular adenomas less than 1 cm in diameter, lacking villous features or high-grade dysplasia. LCRA were defined as adenomas ≥1 cm without features of advanced neoplasia. IMC referred to high-grade neoplasia or carcinoma confined to the muscularis mucosae, without submucosal invasion.

This study was approved by the Ethics Committee of the First Affiliated Hospital of Anhui Medical University (Approval No. PJ 2024-01-33).

Inclusion criteria: (A) Participants met the diagnostic and classification criteria; (B) No antibiotic or microecological treatment had been administered within 3 months prior to enrollment; (C) Written informed consent was obtained from all participants.

Exclusion criteria: (A) History of colorectal cancer or other malignancies; (B) Familial adenomatous polyposis; (C) Antibiotic use within the past 3 months; (D) Prior history of intestinal surgery; (E) Known allergy to bowel cleansing agents; (F) Metastatic colorectal cancer; (G) Inability to comply with study procedures.

### 2.2 DNA extraction from fecal samples

Fresh fecal samples were collected, and DNA was extracted using the TIANamp Soil DNA Kit (spin column type, DP336). The concentration, integrity, and purity of the extracted DNA were assessed using the Agilent 5,400 system (AATI). Only samples that met quality standards were used for subsequent library preparation.

### 2.3 Library construction and metagenomic sequencing

Fecal DNA libraries were prepared using the Rapid Plus DNA Library Prep Kit for Illumina (RK20208). Library quality control included insert size assessment using the AATI system and accurate quantification of effective concentration (≥1.5 nM) using quantitative PCR (qPCR). Sequencing was then performed on the Illumina NovaSeq platform. Raw sequencing data were processed and quality-controlled using fastp software.

### 2.4 Analysis of gut microbiota

Alpha diversity indices, including Chao1, Pielou evenness, Shannon, and Simpson indices, and beta diversity indices, including Bray–Curtis and Jaccard distances, were calculated at the family, genus, and species levels using the vegan package (version 2.6–8). Microbial community composition was visualized through species composition bar plots at multiple taxonomic levels. Comparative analyses of microbial communities among groups were conducted at the phylum, class, order, family, genus, and species levels.

Two complementary approaches were used to identify discriminatory taxa. LEfSe is used to identify individual species with statistically and biologically consistent differences between groups (biomarker discovery), which was performed using the microbiomeMarker package (version 1.9.0). Characteristic species or differential species were defined as linear discriminant analysis (LDA) score > 2.0 and a *p*-value < 0.05. Random forest model was constructed to assess the predictive power of multispecies traits and identify a set of taxa that, when combined, can best classify disease groups, explaining potential interactions and nonlinear relationships. Participants were randomly divided into discovery and test sets in a 7:3 ratio. Feature species selection was conducted using the Boruta algorithm, and the random forest classification model was built using the caret package (version 6.0–94). Functional profiling of the metagenomic data was performed using DIAMOND to align predicted protein sequences against the KEGG and MetaCyc databases for pathway and functional annotation.

### 2.5 Statistical analysis

Between-group differences in alpha diversity were assessed using the Wilcoxon test for two-group comparisons and analysis of variance (ANOVA) for multiple groups. Beta diversity differences were evaluated using PERMANOVA. Differences in bacterial genera between groups were also analyzed using the Wilcoxon test. The diagnostic performance of the random forest model was evaluated using receiver operating characteristic (ROC) curves. A two-sided *p*-value < 0.05 was considered statistically significant.

## 3 Results

### 3.1 Clinical and demographic characteristics

A total of 250 fecal samples were analyzed from five groups: HC (*n* = 30), Polyp (*n* = 52), SCRA (*n* = 58), LCRA (*n* = 56), and IMC (*n* = 54) (Table 1). Patients with IMC were significantly older (*p* for trend < 0.001), had higher body mass index (BMI) (*p* for trend = 0.049), and exhibited the highest prevalence of hypertension (*p* for trend = 0.042). No significant differences were observed among the groups in terms of sex, smoking history, or alcohol consumption (Table 1).

**Table 1:** Relevant clinical characteristics.

Variable		HC	Polyp	SCRA	LCRA	IMC	*F*/*X*^2^	*p*
Cases (*n*)		30	52	58	56	54		
Gender	Male (*n*, %)	18 (60.00%)	34 (65.38%)	36 (62.07%)	38 (67.86%)	41 (75.93%)	χ^2^ = 3.31	0.507
	Female (*n*, %)	12 (40.00%)	18 (34.62%)	22 (37.93%)	18 (32.14%)	13 (24.07%)		
Age (year)		37.63 ± 9.40	50.71 ± 13.44	56.40 ± 10.90	56.07 ± 11.40	57.96 ± 11.92	*F* = 17.84	<0.01
BMI		22.75 ± 2.89	23.85 ± 2.96	24.64 ± 2.97	23.60 ± 2.81	24.33 ± 3.10	*F* = 2.43	0.049
Hypertension History	Yes (*n*, %)	1 (3.33%)	10 (19.23%)	16 (27.59%)	13 (23.21%)	17 (31.48%)	χ^2^ = 9.91	0.042
	No (*n*, %)	29 (96.67%)	42 (80.77%)	42 (72.41%)	43 (76.79%)	37 (68.52%)		
Smoking History	Yes (*n*, %)	5 (16.67%)	12 (23.08%)	7 (12.07%)	18 (32.14%)	16 (29.63%)	χ^2^ = 8.52	0.074
	No (*n*, %)	25 (83.33%)	40 (76.92%)	51 (87.93%)	38 (67.86%)	38 (70.37%)		
Alcohol Consumption	Yes (*n*, %)	3 (10.00%)	12 (23.08%)	8 (13.79%)	13 (23.21%)	15 (27.78%)	χ^2^ = 5.87	0.209
	No (*n*, %)	27 (90.00%)	40 (76.92%)	50 (86.21%)	43 (76.79%)	39 (72.22%)		

BMI, Body mass index, calculated as weight (kg)/height (m)^2^; Normally distributed data are presented as mean ± standard deviation (SD); F, ANOVA; χ^2^, Chi-square test; M, Median; SD, standard deviation.

### 3.2 Alpha and beta diversity

**Figure 1:**
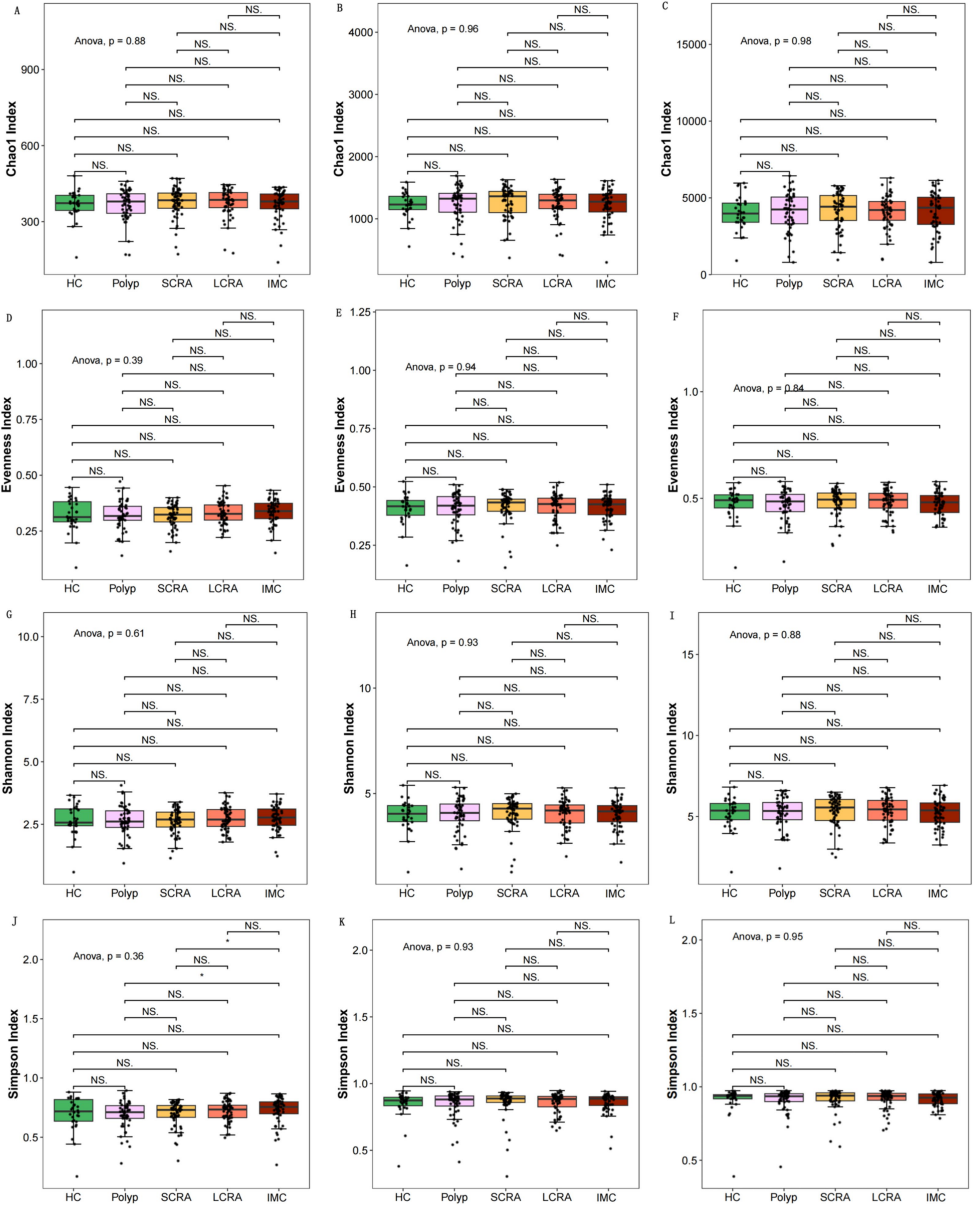
Intergroup comparisons of alpha diversity at different taxonomic levels. Intergroup comparisons of the Chao1 index at the family, genus, and species levels, respectively **(A–C)**. Intergroup comparisons of the Pielou **(D–F)** and the Shannon **(G–I)**. Illustration of the Simpson index **(J–L)**.

Alpha diversity indices (Chao1, Pielou, Shannon, and Simpson) showed no significant differences among the groups at the family, genus, and species levels (Figure 1), suggesting similar microbial richness, evenness, and diversity.

**Figure 2:**
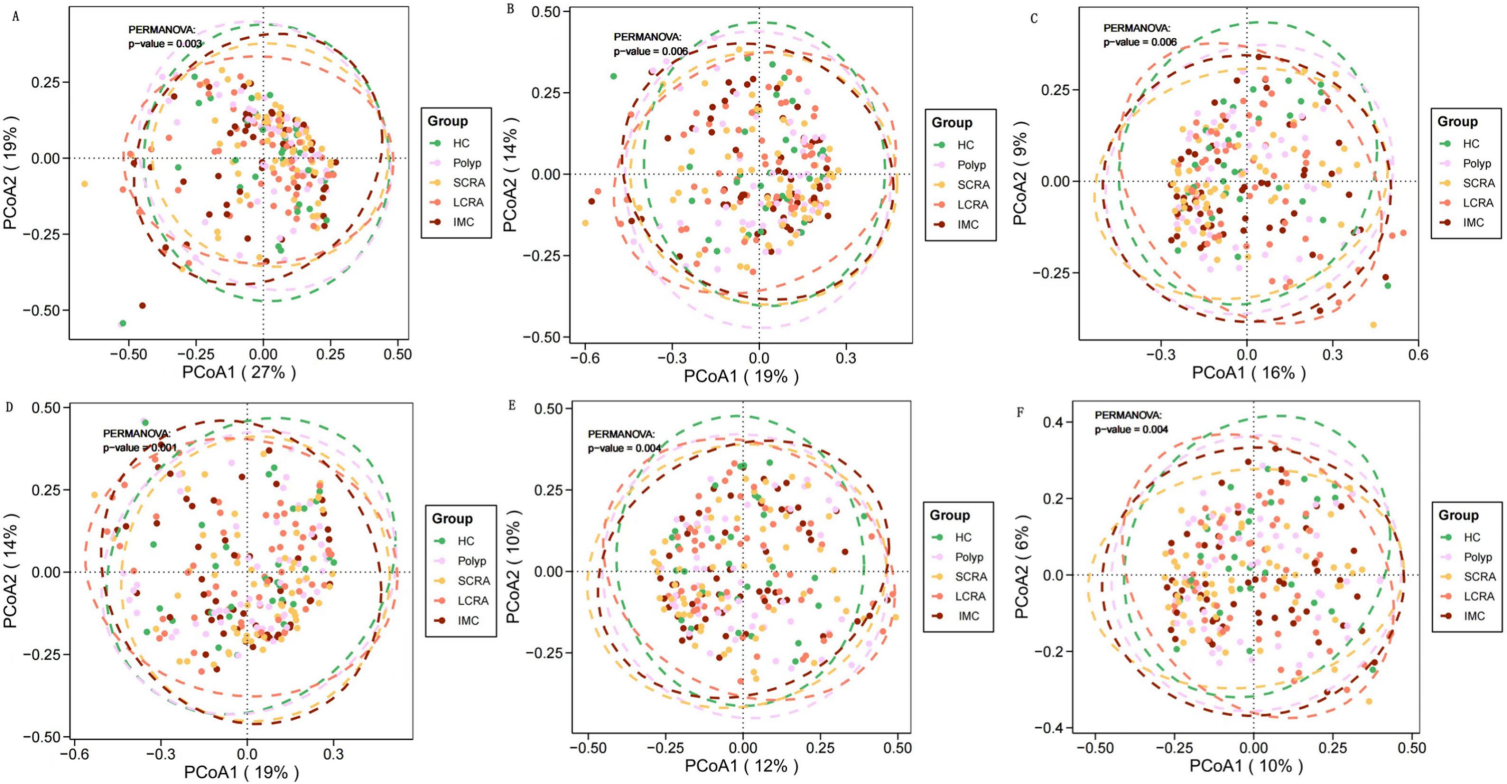
Gut microbiota characteristics of different groups at different taxonomic levels. Principal coordinate analysis (PCoA) of gut microbial composition revealed significant differences among groups at the family, genus, and species levels. PCoA based on Bray-Curtis distance **(A–C)** and Jaccard distance **(D–F)**.

Beta diversity metrics—including Bray–Curtis and Jaccard distances—were calculated and visualized to evaluate microbial community structure at the family, genus, and species levels, using principal coordinates analysis (PCoA). Significant differences in beta diversity were identified among the five groups at all taxonomic levels (Figure 2).

### 3.3 Community composition distribution

**Figure 3:**
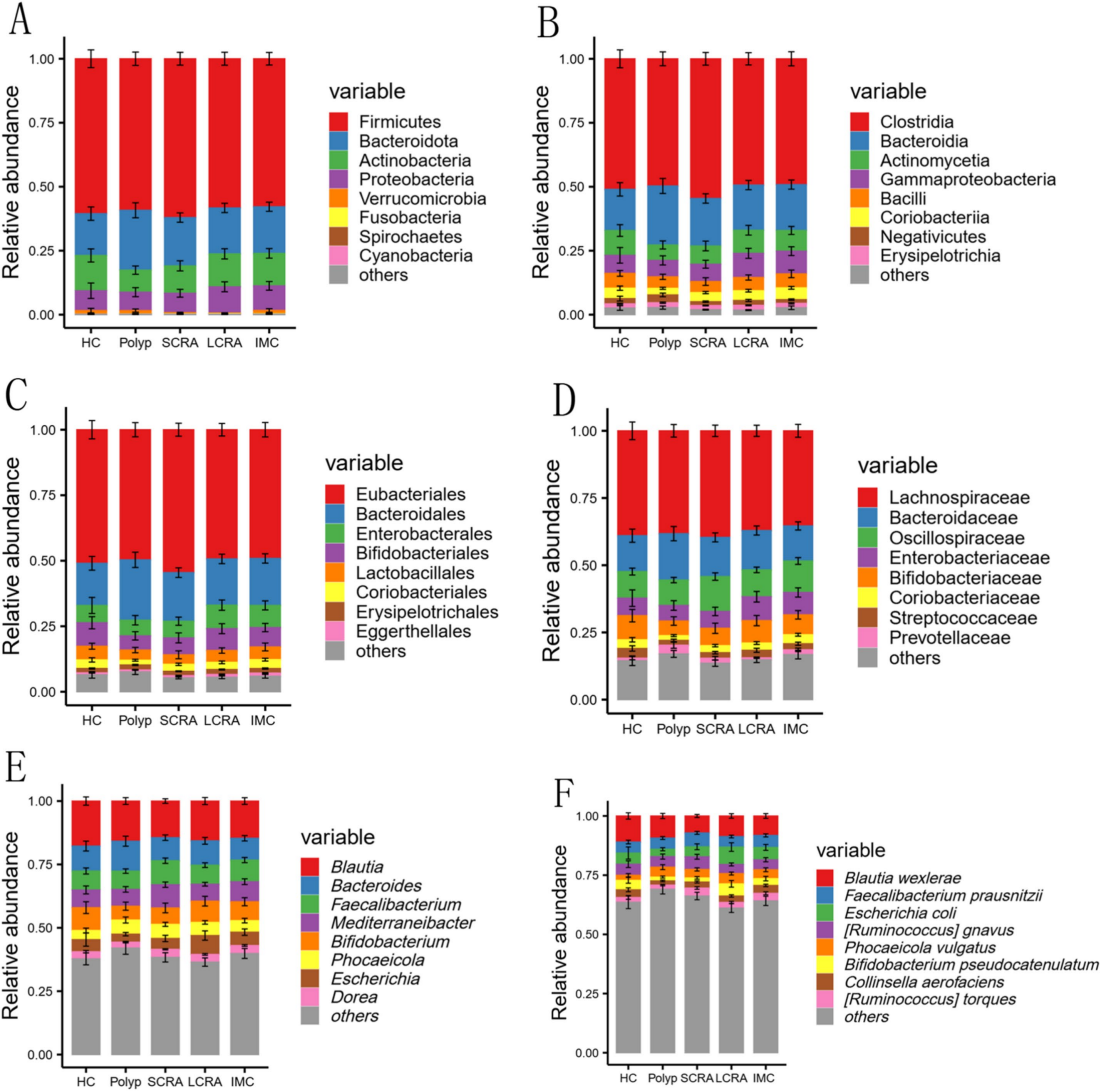
Comparison of gut microbial composition. The distribution of the top nine most abundant taxa at the phylum, class, order, family, genus, and species levels among all groups **(A–F)**.

To further investigate gut microbiota composition, relative abundance was assessed at the phylum, class, order, family, genus, and species levels (Figure 3). Dominant taxa were identified as Bacteroidota at the phylum level, Clostridia at the class level, Eubacteriales at the order level, Oscillospiraceae at the family level, *Mediterraneibacter* at the genus level, and *Blautia wexlerae*, *Escherichia coli*, and *Phocaeicola vulgatus* at the species level (Figure 3).

### 3.4 LEfSe analysis of differential bacterial species

**Figure 4:**
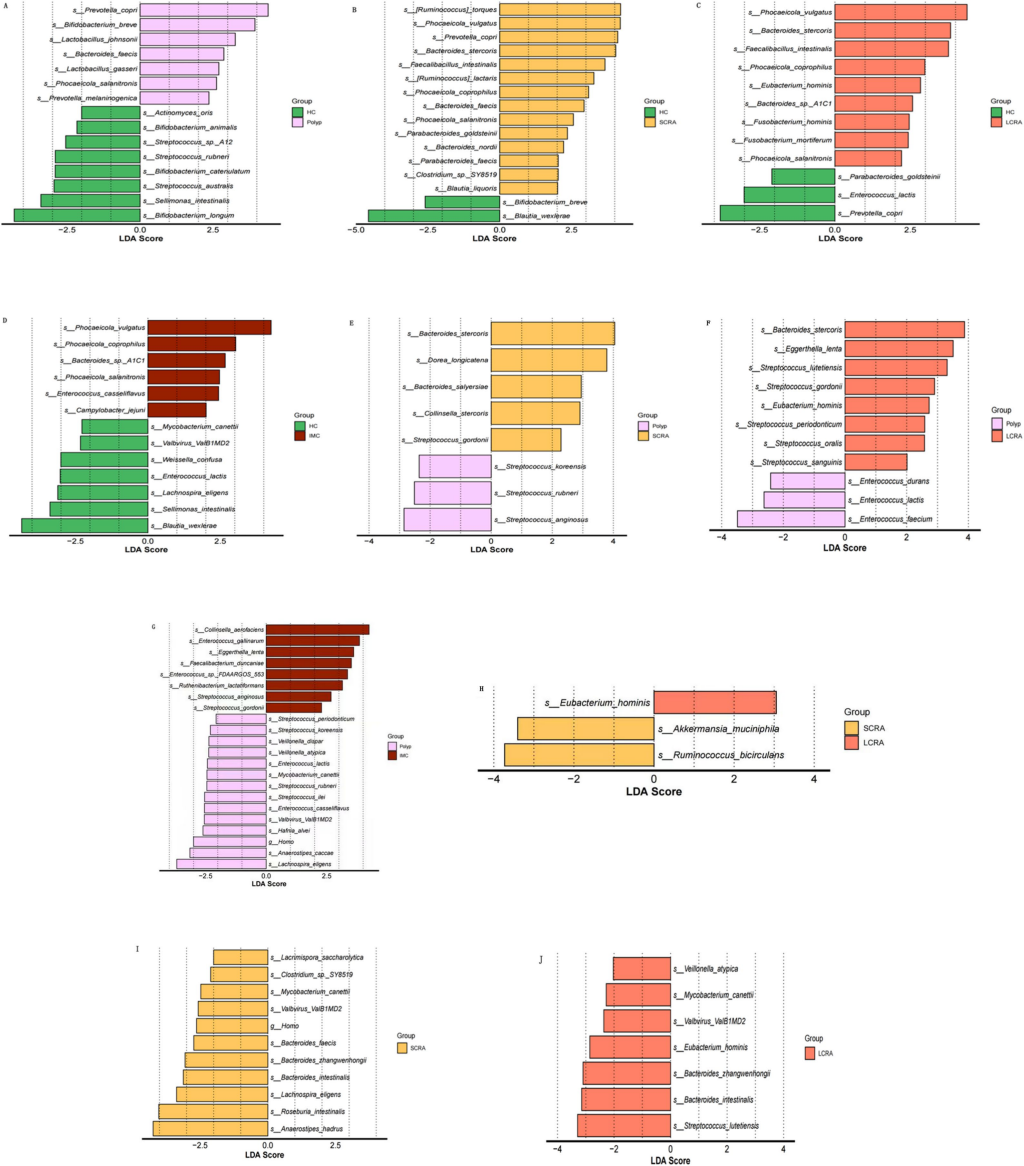
Linear discriminant analysis effect size (LEfSe) identified microbial taxa with significantly relative abundances. Panels **A–J** show pairwise comparisons among groups: **(A)** HC vs. Polyp; **(B)** HC vs. SCRA; **(C)** HC vs. LCRA; **(D)** HC vs. IMC; **(E)** Polyp vs. SCRA; **(F)** Polyp vs. LCRA; **(G)** Polyp vs. IMC; **(H)** SCRA vs. LCRA; **(I)** SCRA vs. IMC; **(J)** LCRA vs.IMC.

LEfSe analysis (LDA score > 3) was performed to identify taxa with significantly different abundances among the five groups (Figure 4). The relative abundances of *Phocaeicola vulgatus* and *Phocaeicola coprophilus* were elevated, while *Sellimonas intestinalis* and *Blautia wexlerae* were reduced in the SCRA, LCRA, and IMC groups. Compared to the Polyp group, *Eggerthella lenta* was more abundant in the LCRA and IMC groups. Conversely, *Bacteroides zhangweihongii* and *Bacteroides intestinalis* exhibited decreased relative abundance in the SCRA and LCRA groups compared to the IMC group. Moreover, *Eubacterium hominis* abundance was significantly increased in the LCRA group than in the SCRA group, whereas *Akkermansia muciniphila* and *Ruminococcus bicirculans* were reduced (Figure 4).

### 3.5 Relative abundance of differential species

**Figure 5:**
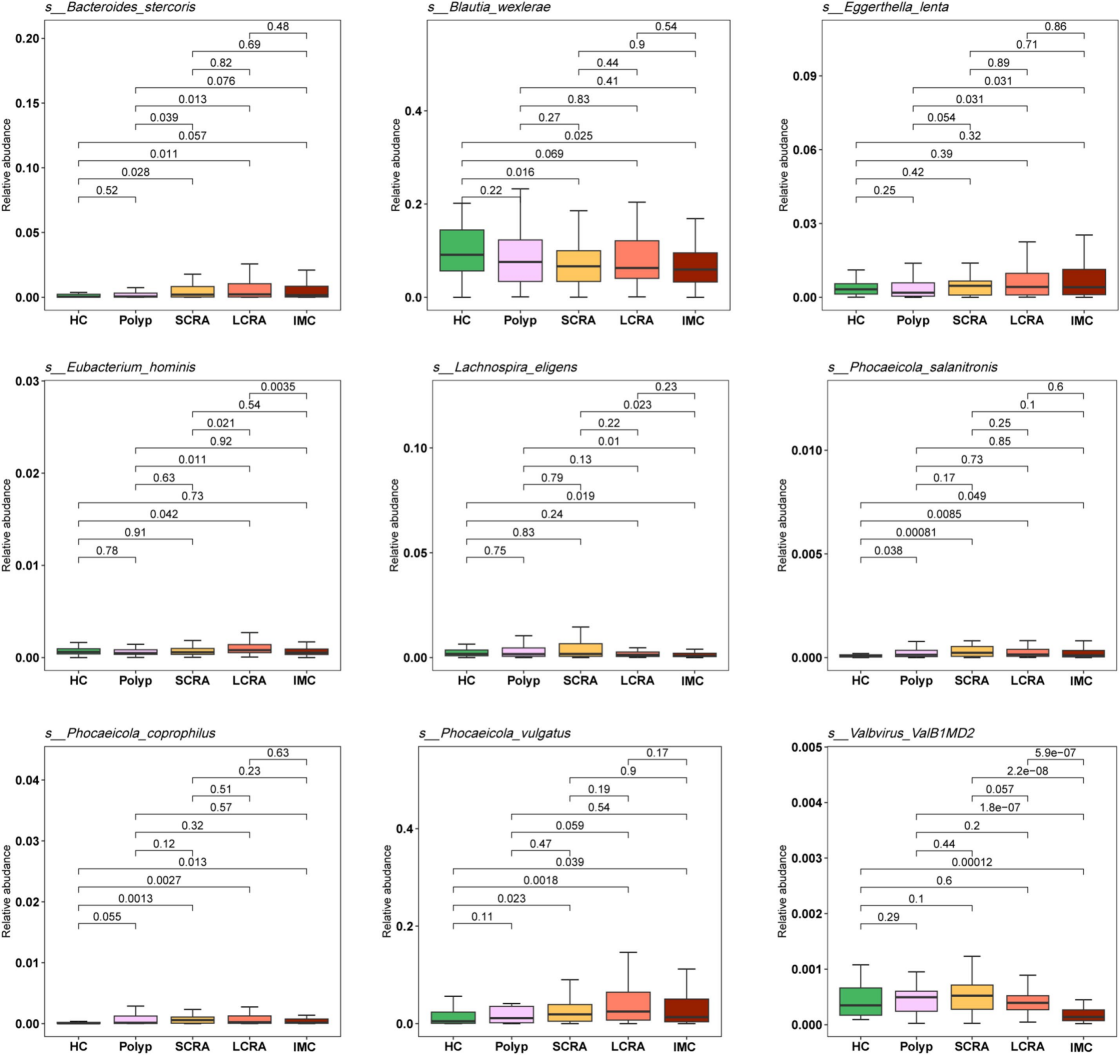
Relative abundances of differentially enriched species identified by LEfSe analysis. The legend indicates sample groups: healthy controls (green), common polyps (pink), small adenomas (yellow), large adenomas (orange), and intramucosal carcinomas (red).

To validate the differences in species abundance identified through LEfSe analysis across the five groups, the Wilcoxon test was performed (Figure 5). Compared to the HC group, the relative abundances of *Phocaeicola vulgatus*, *Phocaeicola coprophilus*, and *Bacteroides stercoris* were significantly increased in the SCRA, LCRA, and IMC groups, whereas *Blautia wexlerae* and *Lachnospira eligens* were significantly decreased in all diseased groups (*p* < 0.05). Additionally, the abundances of *Eubacterium hominis* and *Eggerthella lenta* were significantly elevated in the LCRA group relative to the other groups (*p* < 0.05).

### 3.6 Random forest models identify bacterial species combinations distinguishing disease groups from healthy controls

**Figure 6:**
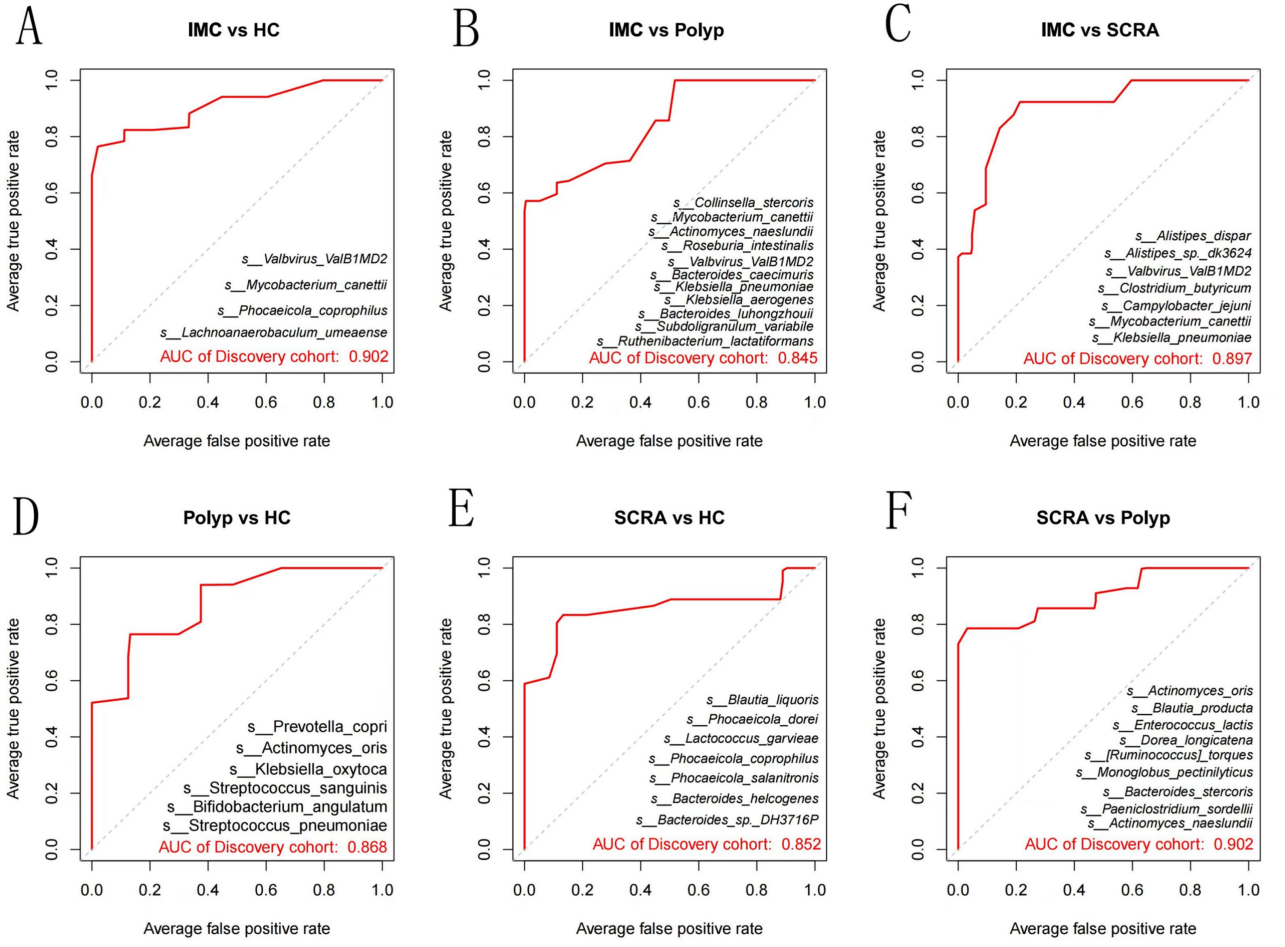
Receiver operating characteristic (ROC) curves at the species level. Panels **A–F** represent pairwise comparisons among groups: **(A)** IMC vs. HC; **(B)** IMC vs. Polyp; **(C)** IMC vs. SCRA; **(D)** Polyp vs. HC; **(E)** SCRA vs. HC; **(F)** SCRA vs. Polyp.

Random forest models were employed to identify combinations of bacterial species capable of distinguishing between different groups (Figure 6). ROC curve analysis demonstrated that these bacterial combinations effectively differentiated IMC from HC (AUC = 0.902), IMC from Polyp (AUC = 0.845), IMC from SCRA (AUC = 0.897), SCRA from HC (AUC = 0.852), SCRA from Polyp (AUC = 0.902), and Polyp from HC (AUC = 0.868). These results indicate that distinct microbial signatures can effectively differentiate the IMC group from other groups.

### 3.7 Potential biological functions

**Figure 7:**
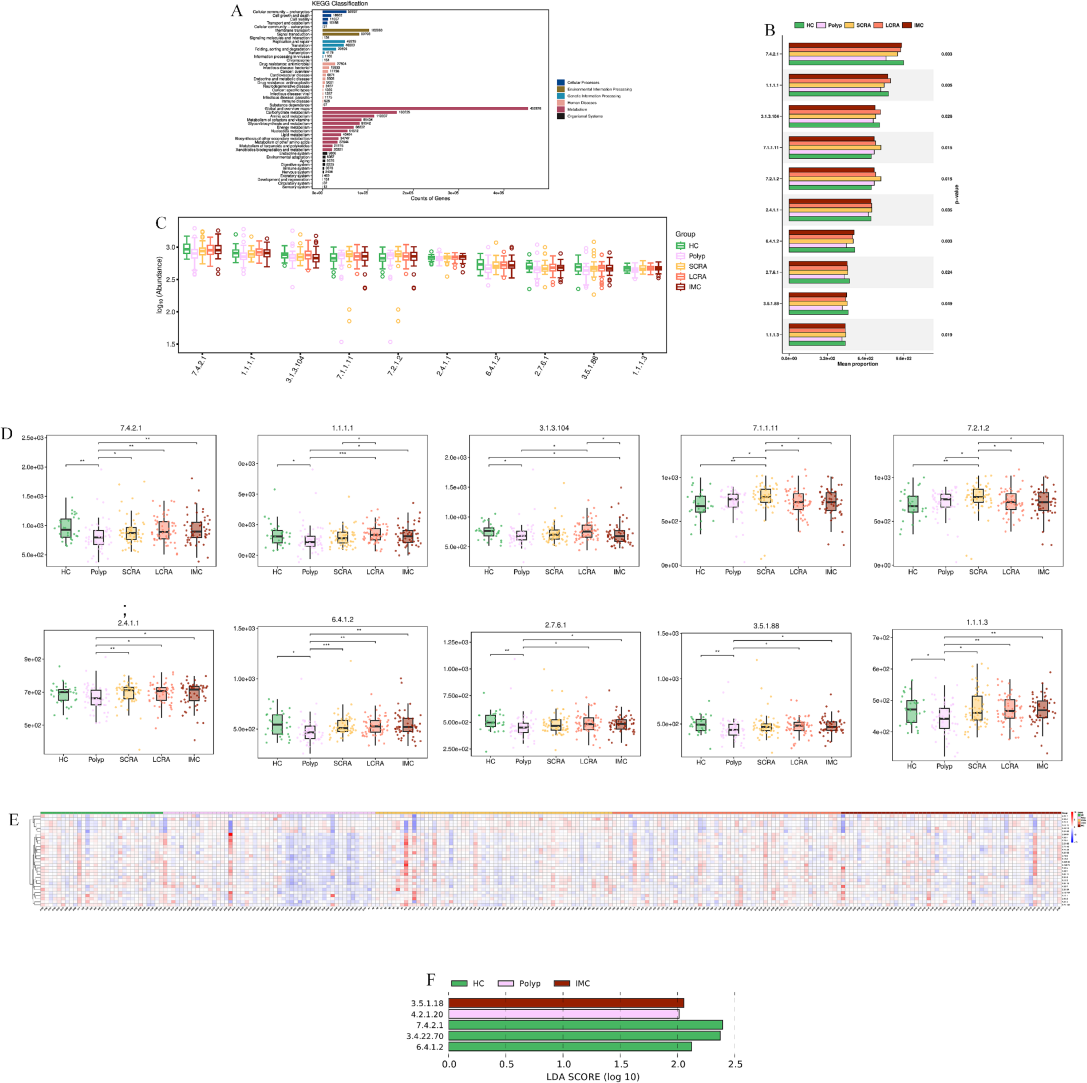
KEGG analysis revealed reprogramming of Microbial Enzyme Activities (EC Numbers). **(A)** KEGG functional annotation of microbial genes. **(B)** Comparison of the average proportion of key Enzyme Commission (EC) numbers. **(C)** Distribution of the relative abundance of selected EC numbers. **(D)** Abundance differences of key enzymes in CRC progression. **(E)** Relative abundance of differentially expressed EC numbers across all samples. **(F)** LEfSe analysis of key enzyme biomarkers distinguishing different stages of CRC.

**Figure 8:**
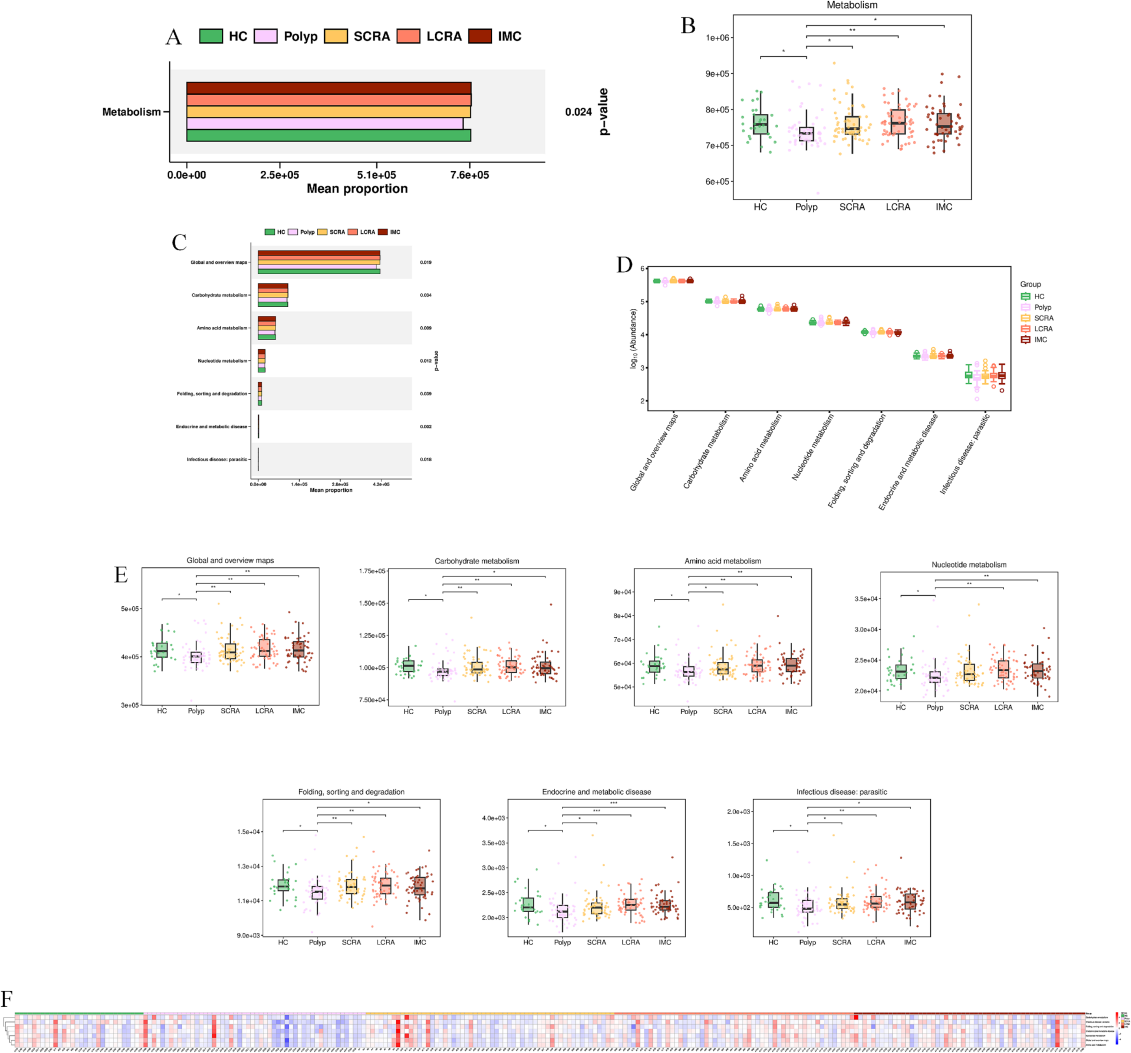
Systematic metabolic upregulation across KEGG Hierarchies at Level 1 and Level 2. **(A)** Mean proportion of the “Metabolism” category (KEGG Level 1). **(B)** Distribution of “Metabolism” category abundance (KEGG Level 1). **(C)** Mean proportion of KEGG Level 2 metabolic subcategories. **(D)** Distribution of the abundance of KEGG Level 2 categories. **(E)** Abundance differences specific of KEGG Level 2 categories. **(F)** Relative abundance of metabolic features across all samples (KEGG Level 2).

**Figure 9:**
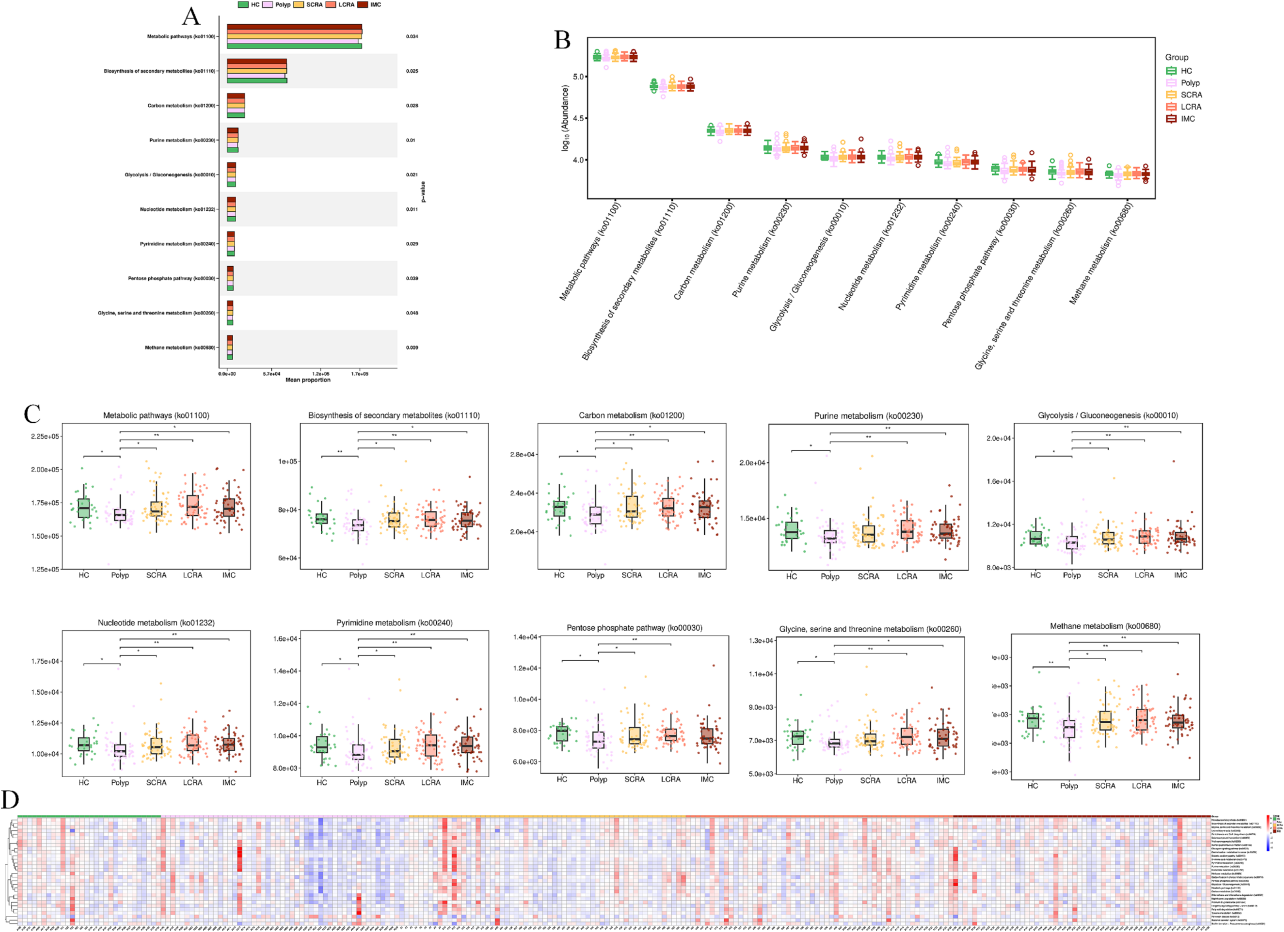
Systematic metabolic upregulation across KEGG Hierarchies at Level3. **(A)** Mean proportion of the “Metabolism” category (KEGG Level 3). **(B)** Distribution of the abundance of KEGG Level 3 metabolic pathways. **(C)** Abundance differences of specific KEGG Level 3 metabolic pathways. **(D)** Relative abundance of differentially expressed metabolic pathways.

KEGG annotation linked predominant gut bacterial taxa to six core functional categories, while Kruskal-Wallis tests of EC numbers identified functional reprogramming across the CRC progression, marked by key shifts in energy metabolism, quorum-quenching activity, and amino acid synthesis-related enzyme dynamics (Figure 7). Systematic metabolic upregulation was observed across KEGG hierarchies: at Level 1, “Metabolism” capacity increased significantly with CRC progression, linking global microbial metabolism to disease advancement (*p* < 0.05, Figures 8A,B); at Level 2, this upregulation was driven by activated central metabolic, carbohydrate, and nucleotide metabolism, with late-stage enrichment of “Infectious Diseases: Parasitic” pathways also noted (Figures 8C–F); at Level 3, specific pathways including carbon metabolism, purine metabolism, the pentose phosphate pathway, and glycine/serine/threonine metabolism were consistently upregulated, clearly indicating a functional shift toward microbial biosynthetic processes (Figure 9).

**Figure 10:**
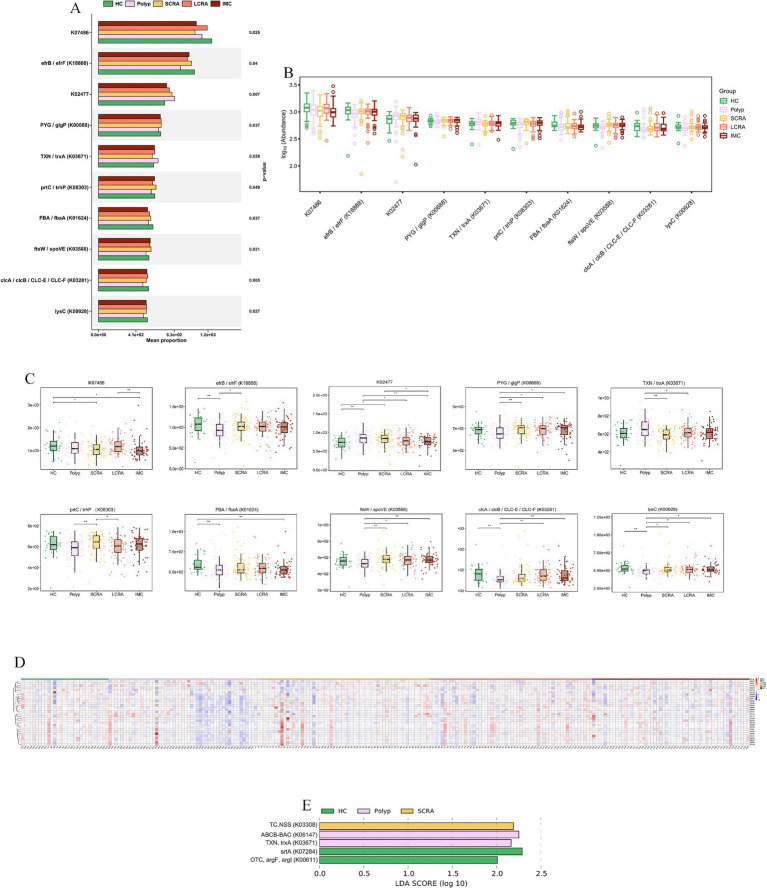
KEGG KO analysis confirmed a hyper-proliferative phenotype at Gene-Level. **(A)** Functional classification of KEGG KO genes. **(B)** Abundance distribution of KEGG KO genes. **(C)** Abundance differences of key KEGG KO genes. **(D)** Relative abundance of differentially expressed KEGG KO genes across all samples.

KEGG Orthology (KO) analysis confirmed coordinated upregulation of genes involved in DNA replication/repair (e.g., K00763, pncB), stress response (e.g., K03671, trxA), and primary metabolism (e.g., K00928, lysC); heatmap analysis further validated that this functional upregulation was extensive and synchronized in intramucosal carcinoma (Figure 10, Supplementary Table S3).

**Figure 11:**
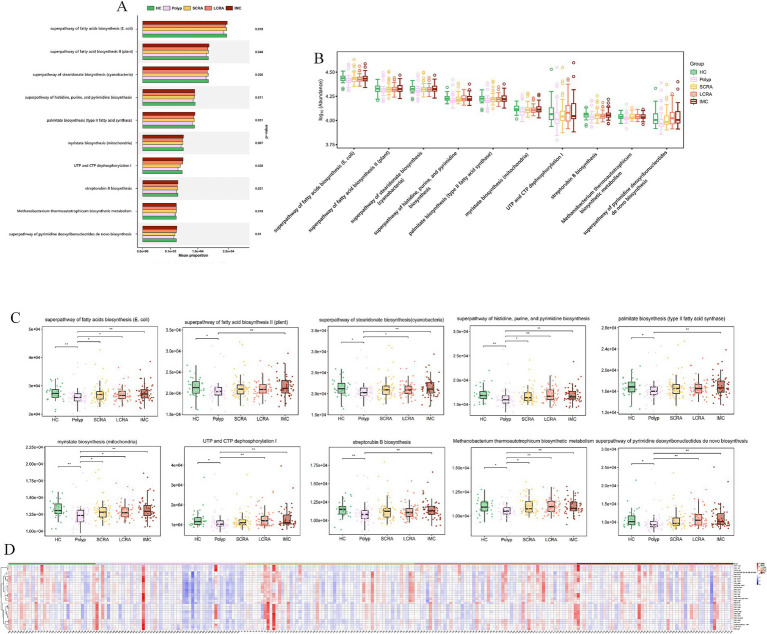
MetaCyc. pathway analysis revealed loss of protective and commensal metabolic pathways. **(A)** Mean proportion of MetaCyc pathways across groups. **(B)** Distribution of the abundance of MetaCyc pathways. **(C)** Abundance differences of key MetaCyc pathways. **(D)** Relative abundance of differentially expressed MetaCyc pathways.

**Figure 12:**
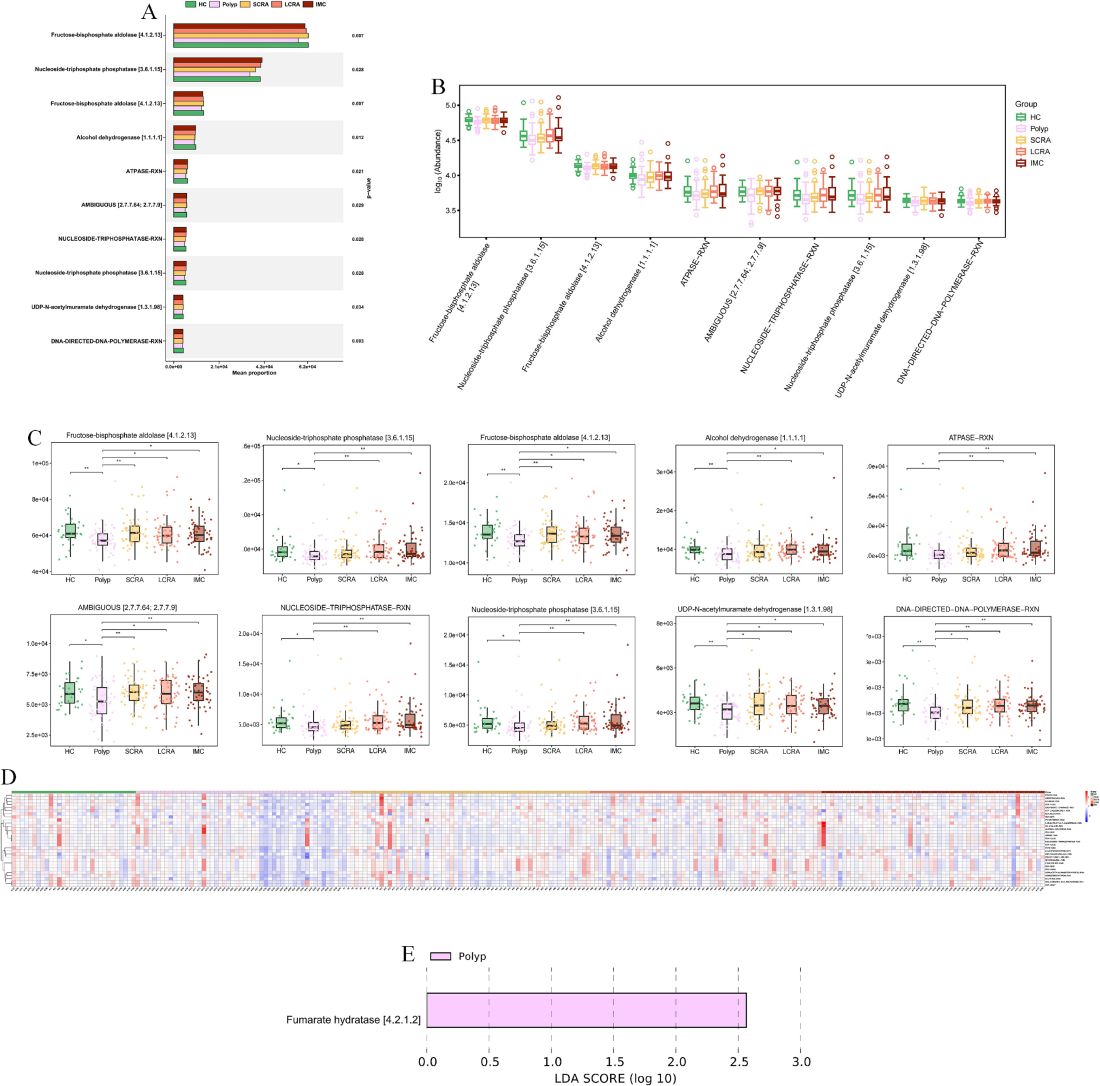
MetaCyc. reaction indicated systemic impairment of energy and biosynthetic metabolism. **(A)** Mean proportion of MetaCyc reactions across groups. **(B)** Abundance distribution of MetaCyc reactions. **(C)** Abundance differences of key MetaCyc reactions. **(D)** Relative abundance of differentially expressed MetaCyc reactions. **(E)** LEfSe analysis of key MetaCyc reaction biomarkers distinguishing different stages of CRC.

MetaCyc analyses revealed functional remodeling of the gut microbiome across the CRC progression. Pathway analysis showed that the abundance of protective microbial pathways (such as PWY-6285, *E. coli* fatty acid biosynthesis; PWY-7596, stearidonate biosynthesis; PWY1A0–6,120, streptorubin B biosynthesis; and PWY-6146, Methanobacterium biosynthesis) was reduced in the CRC continuum compared with healthy controls (Figure 11, Supplementary Table S4). Meanwhile, reaction analysis demonstrated systemic impairment in microbial energy metabolism (e.g., ATPASE-RXN), biosynthetic metabolism (e.g., UDPNACETYLMURAMATEDEHYDROG-RXN [UDP-N-acetylmuramate dehydrogenase; EC 1.3.1.98] and GLUC1PURIDYLTRANS-RXN [ambiguous; EC 2.7.7.64/2.7.7.9]), and replication (e.g., DNA-DIRECTED-DNA-POLYMERASE-RXN) (Figure 12, Supplementary Table S5). These changes collectively reflect global functional reprogramming of the gut microbiome.

## 4 Discussion

Gut microbiota dysbiosis plays a pivotal role in the development and progression of CRC. Comprehensive investigations into the gut microbiota and its associations with the pathological evolution of CRC may offer valuable insights for its prevention and treatment (Jackson and Theiss, 2020). In this study, MS was employed to investigate gut microbiota dynamics along the adenoma–carcinoma sequence, encompassing multiple pathological subgroups, including polyp, SCRA, LCRA, and IMC. Additionally, quantitative analyses were conducted to identify potential microbial biomarkers and explore associations between the gut microbiota and CRC progression, using *α*-diversity, *β*-diversity, and LEfSe analyses.

No significant differences in α-diversity indices were observed among the groups, which is consistent with previous studies. Potential contributing factors include the complexity of host variables, microbial ecosystem characteristics, and limitations in detection techniques and sample processing (Janney et al., 2020; Song et al., 2020). In contrast, significant differences in β-diversity were observed across the groups at the family, genus, and species levels. While some studies have reported significant β-diversity differences only at the family and genus levels between HC and CRC groups (Cheng et al., 2020), such variations may be attributed to increased pro-inflammatory bacterial populations, reductions in beneficial bacteria due to intestinal microenvironmental shifts (Xia et al., 2023), and differences in host immune status (Fusco et al., 2023).

Species composition analysis revealed stage-specific variations across all taxonomic levels. At the phylum level, the polyp group exhibited a higher abundance of Bacteroidota and a lower abundance of Actinobacteria. Bacteroidota, known for its strong polysaccharide metabolic capacity, may exhibits competitive expansion under low dietary fiber conditions (Lam et al., 2023). High-fat/high-protein diets, as well as inflammatory intestinal conditions, further promote its dominance. Conversely, a deficiency in dietary fiber may reduce Actinobacteria abundance, while high-fat/high-protein diets could inhibit its growth via gut pH alteration and redox potential shifts (Colella et al., 2023).

An increased abundance of Proteobacteria was observed in the LCRA and IMC groups, possibly reflecting an altered redox potential that favors the growth of facultative anaerobes (Wu et al., 2023). Moreover, immunosuppressive conditions associated with colorectal adenoma and carcinoma may also contribute to Proteobacteria proliferation (Feizi et al., 2023).

At the family level, the SCRA group displayed an increased abundance of Oscillospiraceae and a decreased abundance of Enterobacteriaceae. Oscillospiraceae may gain a competitive advantage in the early adenoma microenvironment by fermenting dietary fiber and producing short-chain fatty acids (SCFAs), which play roles in regulating host metabolism and immunity (Liu et al., 2023). In contrast, Enterobacteriaceae may be disadvantaged by their reduced adaptability to local redox conditions, limited nutrient acquisition efficiency, and suboptimal immune evasion mechanisms (Quaglio et al., 2022).

At the genus level, the SCRA group showed increased *Mediterraneibacter*, while the LCRA group exhibited higher *Bifidobacterium* abundance. The immune microenvironment of early adenomas appears to be more permissive toward *Mediterraneibacter*, whereas *Bifidobacterium* may adapt to immune changes by modulating local immune responses in more advanced lesions (Cai et al., 2022; Karpiński et al., 2022). Notably, the abundance of *Escherichia* was decreased in the SCRA group but increased in the LCRA group, suggesting a role in adenoma progression. Previous studies have indicated that high-fat/high-protein diets lead to the production of intermediate metabolites such as branched-chain amino acids (BCAAs) and specific fatty acids, which serve as carbon and nitrogen sources for *Escherichia*, thereby promoting its proliferation (Hou et al., 2022).

Furthermore, *Escherichia*-derived endotoxins can activate the NF-κB pathway, enhancing local inflammation and facilitating abnormal proliferation and differentiation of adenomatous cells (Kvakova et al., 2022). Particularly, pathogenic strains like *Escherichia coli* (EPEC), may enhance adenoma invasiveness and drive disease progression by modulating host signaling pathways through the type III secretion system (Clay et al., 2022).

In this study, LEfSe analysis revealed significant enrichment of *Phocaeicola vulgatus* and *Phocaeicola coprophilus* in the SCRA, LCRA, and IMC groups, particularly in the LCRA group (Supplementary Table S1). Existing evidence implicates *Phocaeicola* species are associated with intestinal immune regulation and colorectal cancer progression. *Phocaeicola vulgatus* promotes the adenoma–carcinoma transition by inducing M2 macrophage polarization and activating the NF-κB pathway (Welham et al., 2023; Ala, 2022). *Phocaeicola coprophilus* evades immune clearance by exploiting the immunosuppressive effects of regulatory T cells (Tregs) and gains a proliferative advantage through the metabolic utilization of lactic acid in the tumor microenvironment (Shen et al., 2022).

Our findings also demonstrated that the abundance of *Blautia wexlerae*, a bacterium belonging to the phylum Firmicutes, was significantly decreased in the SCRA, LCRA, and IMC groups compared with the HC group, in line with previous reports (Supplementary Table S1). The beneficial roles of Firmicutes have been well documented, and their decreased abundance has been associated with esophageal cancer, lung cancer, and type 2 diabetes (Lee et al., 2023).

One original finding of this study is that the abundance of *Sellimonas intestinalis* was significantly reduced in the Polyp and IMC groups relative to the HC group, whereas no significant change was observed in the SCRA and LCRA groups. This stage-specific pattern may reflect microbial competition dynamics (Supplementary Table S1). In the common polyp and IMC groups, a marked microbial imbalance was present, enabling more adaptive pathogenic bacteria (e.g., facultative anaerobes such as Enterobacteriaceae) to outcompete *Sellimonas intestinalis* for resources. In contrast, the adenoma groups maintained a relatively stable microbial environment with reduced competitive pressure, thereby preserving the abundance of *Sellimonas intestinalis* (Romanov et al., 2022). Nevertheless, due to limitations in sample size and specimen conditions, these findings require validation through larger-scale studies.

LEfSe analysis further identified significantly elevated *Eggerthella lenta* abundance in the LCRA and IMC groups compared with the Polyp group, with no significant increase in the SCRA group (Supplementary Table S1). MS and animal model studies have similarly shown increased abundance of *Eggerthella lenta* with disease progression in advanced adenoma and CRC patients (Lee et al., 2023; Romanov et al., 2022), suggesting a potential role in the malignant transformation of inflammatory polyps. During the early adenoma stage, only mild immunosuppression and minimal microbial dysbiosis are present. However, in advanced adenoma and IMC stages, decreased immune surveillance and reduced probiotic populations relieve competitive inhibition, allowing *Eggerthella lenta* to increase in abundance. This bacterium may further exacerbate disease progression by inducing epithelial–mesenchymal transition (EMT) and promoting metabolic reprogramming, thereby establishing a vicious cycle that enhances tumor invasiveness (Avelar-Barragan et al., 2022; Zhang et al., 2021).

The abundances of *Bacteroides zhangwenhongii* and *Bacteroides intestinalis* were significantly decreased in the SCRA and LCRA groups compared with the IMC group, suggesting their potential involvement in the adenoma–carcinoma transition (Supplementary Table S1). Clos-Garcia et al. reported abnormal levels of *Bacteroides intestinalis* in patients with colorectal adenoma and CRC (Clos-Garcia et al., 2020). Furthermore, animal experiments have confirmed that *Bacteroides intestinalis* can promote tumorigenesis in AOM-DSS-induced mice by regulating inflammation- and apoptosis-related gene expression (Liu et al., 2020). Since the role of *Bacteroides zhangwenhongii* in CRC remains unclear, further studies are needed to elucidate its mechanistic involvement.

In the LCRA group, *Eubacterium hominis* was increased, whereas *Akkermansia muciniphila* and *Ruminococcus bicirculans* were decreased (Supplementary Table S1). The increased abundance of *Eubacterium hominis* in patients with advanced adenomas may alter the intestinal short-chain fatty acid (SCFA) profile (e.g., butyrate) by competing for metabolic substrates required by SCFA-producing microorganisms. This competition reduces the availability of substrates for beneficial bacteria, indirectly impairing the physiological function of intestinal epithelial cells and promoting adenoma progression (Cheng et al., 2020).

Random forest model analysis revealed significant microbiota disparities across several group comparisons (e.g., SCRA vs. Polyp, IMC vs. HC). The gut microbiota-based diagnostic models exhibited high predictive performance (AUC ≥ 0.8), suggesting that gut microbial profiles along the polyp–adenoma–carcinoma axis may serve as reliable tools for differential diagnosis. Nevertheless, these results warrant further validation through larger, multicenter investigations.

The key species identified by LEfSe were not the same as the top-level characteristics of the random forest model. This is to be expected because the two approaches solve different problems. LEfSe identifies a single taxa with the largest effect size, while a random forest selects a combination of features that work together to maximize prediction accuracy, even if some individual features have only moderate effects. This highlights that CRC progression is associated with strong individual bacterial signaling and complex multispecies community shifts. Future models could explore the capabilities of combining both approaches to potentially improve performance.

The progression of CRC may not follow a linear trajectory. Potential “tipping points” could exist, for instance, the transition from health to polyp represents one critical shift, while the advancement from late colorectal adenoma (LCRA) to intramucosal carcinoma (IMC) constitutes another drastic alteration, with the intermediate phase (sessile serrated lesions, SCRA) possibly maintaining relative stability. Crucially, changes in species composition do not directly equate to linear functional alterations. In early stages, reductions in beneficial bacteria might be compensated by increased abundance of functionally redundant taxa, preserving functional homeostasis. Functional collapse likely occurs only when dysbiosis surpasses a critical threshold. At specific phases, host-derived drivers such as immune responses and inflammatory states may override microbial influences, generating complex fluctuations in the microbiota rather than linear progression.

We noticed that the IMC group exhibited significant differences in age, BMI, and hypertension status compared to other groups in this study. As reported, age-related declines in anaerobic bacteria like *Bifidobacterium* have been observed, resulting in the low systemic inflammatory status and malnutrition in older adults (Rinninella et al., 2019). While obesity disrupts the microbiota-host metabolic balance by reducing butyrate-producing bacteria (e.g., *Akkermansia*, *Faecalibacterium*) and expanding pro-inflammatory taxa (e.g., Enterobacteriaceae, Alistipes) (Gerard, 2016). These factors are established independent factors of gut dysbiosis and may confound the interpretation of microbiota changes attributed solely to the cancer stage. Additionally, the sample size distribution across groups was uneven, and significant intergroup differences in age and BMI may confound microbiota comparisons. Participants with normal intestinal mucosa confirmed by colonoscopy were enrolled in the healthy control group. Those with inflammatory findings, polyps, previous colorectal surgery, or allergies to bowel preparation agents were excluded. Due to these stringent inclusion criteria, recruitment of eligible healthy control subjects proved challenging, resulting in a relatively small sample size for this group. To statistically account for these confounders, we employed generalized linear models (GLMs) to adjust for age, BMI, and hypertension. Our analysis revealed that while the significance of certain microbial taxa changed after adjustment, the overall trends remained consistent, thus supporting the robustness of our primary findings. The results of the multifactor adjustment can be found in Supplementary Table S2. Larger multicenter cohorts and analyses with multivariate adjustments are required to validate the robustness of our findings.

This study systematically examined gut microbiota differences among five defined groups, identifying key microbial signatures along the “colorectal polyp–adenoma–carcinoma” axis. Compared with previous studies, this work further stratified colorectal polyp subtypes and analyzed the corresponding variations in intestinal microbiota. Subgroup analysis revealed significant differences in the abundances of *Phocaeicola vulgatus*, *Phocaeicola coprophilus*, and *Sellimonas intestinalis*, while *Bacteroides zhangwenhongii* and *Bacteroides intestinalis* were identified as potential contributors to the adenoma–carcinoma transition. Moreover, random forest-based prediction models demonstrated robust diagnostic performance. However, this study has several limitations. We used shotgun metagenomic sequencing to obtain detailed insights into microbial identification, which can get achieve more detailed taxonomic resolution and functional profiling, such as metabolic pathway annotation via HUMAnN3, MetaCyc, or KEGG compared with 16S rRNA sequencing. Although it theoretically enables detection of fungal and viral genomes, our study exclusively reported bacterial profiles. This limitation may arise from insufficient fungal/viral DNA for robust detection and bioinformatic filtering to exclude non-bacterial reads. Further, integrating both metagenomic sequencing and 16S rRNA sequencing might enable more efficient and accurate characterization of microbial community composition, diversity, and functional potential.

KEGG analyses of gut microbiota showed CRC drives microbial metabolic remodeling into a pro-tumorigenic phenotype. Elevated overall metabolic capacity (KEGG Level 1) suggests hyperactive microbes accumulate in the tumor microenvironment (Luo et al., 2025), potentially producing genotoxic/pro-inflammatory metabolites to promote tumor progression. Key Enzyme Commission (EC) activity shifts mark CRC: energy metabolism adapts to tumor demands, early loss of quorum-quenching function may expand pro-carcinogenic taxa (Waheed et al., 2023), and a V-shaped essential amino acid synthesis pattern (EC 1.1.1.3, homoserine dehydrogenase) reflects microbial adaptation. Coherent KEGG upregulation confirms system-wide activation, with enhanced central/carbohydrate/nucleotide metabolism and upregulated DNA replication/stress response genes supporting microbial survival (Cheng et al., 2025), aligning with adenoma-carcinoma microbial shifts.

MetaCyc analyses revealed further CRC-associated reprogramming: lost protective pathways (e.g., PWY-6285, superpathway of *E. coli* fatty acid biosynthesis; PWY-7596, superpathway of cyanobacterial stearidonate biosynthesis) signal ecosystem dysfunction, correlating with stage-specific signatures (e.g., depleted *Blautia wexlerae*). For highlighted pathways: downregulated glycolytic/energy enzymes impair short-chain fatty acid (SCFA) production; reduced alcohol dehydrogenase disrupts bile acid detoxification; nucleotide metabolism disruption impacts polyamine synthesis; impaired sugar nucleotide synthesis drives microbial mucin degradation.

Observed changes reflect systematic functional collapse: energy metabolism shifts from ordered energy storage and mobilization (EC 2.4.1.1, glycogen phosphorylase, 1,4-*α*-D-glucan:phosphate α-D-glucosyltransferase) to sustained high-rate energy production and consumption (EC 7.1.1.11, ferredoxin: NAD^+^ oxidoreductase [H^+^-transporting], Rnf complex) then fails, and coordinated downregulation lowers microbial proliferative capacity. We identified key CRC-microbiota alterations, offering biological insights and potential biomarkers. Targeted DIAMOND profiling will quantify highlighted pathways (SCFAs, bile acids, polyamines, mucin degradation) to validate microbial contributions to CRC.

Future mechanistic investigations and functional validations are needed to clarify the pathogenic roles of key taxa identified. And future studies should integrate comprehensive functional metagenomic and metabolomic analyses to gain deeper insights into the microbial contributions to colorectal carcinogenesis. As a result, our interpretations remain correlative and lack direct functional validation. Future studies should integrate comprehensive functional metagenomics with metabolomics analyses to elucidate the mechanistic roles of the microbiota in colorectal carcinogenesis. Furthermore, mechanistic investigations and functional validations are necessary to clarify the pathogenic contributions of the key microbial taxa identified. Additionally, it did not explore interactions among microbiota, host metabolism, and immune factors, nor did it fully elucidate the roles of *Bacteroides zhangwenhongii* and *Bacteroides intestinalis* in colorectal carcinogenesis. Future multicenter studies with large cohorts are needed to clarify the pathogenic mechanisms of these bacteria and to further delineate the relationship between gut microbiota and CRC. Finally, our hypothesis on the functional implications and potential roles of specific taxa lacks sufficient supporting evidence. Furthermore, although the random forest model achieved high AUC values, its generalizability was not fully validated, as k-fold cross-validation or external validation was not conducted. Future studies with independent validation cohorts are needed to confirm the robustness of these microbial diagnostic signatures.

In conclusion, the gut microbiota, particularly its community structure, exhibits significant differences between healthy individuals and patients with various stages of colorectal lesions. Specific combinations of bacterial species, identified through random forest modeling, can effectively distinguish the IMC group from other groups. Alterations in the gut microbiota along the “polyp–adenoma–carcinoma” axis may drive lesion progression, thereby providing a foundation for early risk assessment, diagnosis, and therapeutic interventions in colorectal cancer.

## Data Availability

The datasets presented in this article are not readily available because follow-up analyses are ongoing. Requests to access the datasets should be directed to the corresponding author.
